# Timing of syncope in ictal asystole as a guide when considering pacemaker implantation

**DOI:** 10.1111/jce.15239

**Published:** 2021-09-19

**Authors:** Anouk van Westrhenen, Sharon Shmuely, Rainer Surges, Beate Diehl, Daniel Friedman, Frans S. S. Leijten, Jorien van Hoey Smith, David G. Benditt, J. Gert van Dijk, Roland D. Thijs

**Affiliations:** ^1^ Stichting Epilepsie Instellingen Nederland (SEIN) Zwolle The Netherlands; ^2^ Department of Neurology and Clinical Neurophysiology Leiden University Medical Center (LUMC) Leiden The Netherlands; ^3^ NIHR University College London Hospitals Biomedical Research Centre UCL Queen Square Institute of Neurology London United Kingdom; ^4^ Department of Epileptology University of Bonn Medical Center Bonn Germany; ^5^ NYU Department of Neurology New York New York USA; ^6^ Department of Neurophysiology University Medical Center Utrecht (UMCU) Utrecht The Netherlands; ^7^ Cardiovascular Division, Cardiac Arrhythmia Center University of Minnesota Medical School Minneapolis Minnesota USA

**Keywords:** autonomic nervous system, blood pressure, epilepsy, focal seizure(s), transient loss of consciousness (TLOC), vasodepression

## Abstract

**Introduction:**

In patients with ictal asystole (IA) both cardioinhibition and vasodepression may contribute to syncopal loss of consciousness. We investigated the temporal relationship between onset of asystole and development of syncope in IA, to estimate the frequency with which pacemaker therapy, by preventing severe bradycardia, may diminish syncope risk.

**Methods:**

In this retrospective cohort study, we searched video‐EEG databases for individuals with focal seizures and IA (asystole ≥ 3 s preceded by heart rate deceleration) and assessed the durations of asystole and syncope and their temporal relationship. Syncope was evaluated using both video observations (loss of muscle tone) and EEG (generalized slowing/flattening). We assumed that asystole starting ≤3 s before syncope onset, or after syncope began, could not have been the dominant cause.

**Results:**

We identified 38 seizures with IA from 29 individuals (17 males; median age: 41 years). Syncope occurred in 22/38 seizures with IA and was more frequent in those with longer IA duration (median duration: 20 [range: 5–32] vs. 5 [range: 3–9] s; *p* < .001) and those with the patient seated vs. supine (79% vs. 46%; *p* = .049). IA onset always preceded syncope. In 20/22 seizures (91%), IA preceded syncope by >3 s. Thus, in only two instances was vasodepression rather than cardioinhibition the dominant presumptive syncope triggering mechanism.

**Conclusions:**

In IA, cardioinhibition played an important role in most seizure‐induced syncopal events, thereby favoring the potential utility of pacemaker implantation in patients with difficult to suppress IA.

## 1 INTRODUCTION

Ictal asystole (IA) is a seizure manifestation affecting 0.3%–0.4% of people with refractory focal epilepsy admitted for video‐EEG monitoring, and mostly occurs in the context of temporal lobe epilepsy.^1^, ^2^ IA appears to occur exclusively in focal impaired awareness seizures, and is often misdiagnosed as a primary cardiologic phenomenon due to ECG documentation of marked bradyarrhythmia. Seizure‐induced asystole may, therefore, be considerably underreported and a substantial proportion of people with IA may not receive optimal treatment.^3^, ^4^, ^5^

It is thought that IA seizures are self‐limited as the resulting global cerebral ischemia induced by the asystole ends the seizure.^1^, ^2^, ^4^, ^6^ Nonetheless, dangerous traumatic falls may occur due to sudden loss of muscle tone.^7^ Consequently, treatment is essential, and primary treatment should focus on optimizing seizure control with antiseizure medication or if necessary epilepsy surgery.^5^, ^7^, ^8^, ^9^ However, pacemaker implantation may be considered if the primary treatment approach fails.

The mechanism of syncopal loss of consciousness (LOC) in IA is believed to be similar to that of reflex syncope, involving overactivity of autonomic reflex pathways.^7^, ^10^, ^11^ In reflex syncope, cardioinhibitory (i.e., vagal lowering of heart rate), as well as vasodepressive (i.e., blood pressure [BP] lowering independent of heart rate) pathways together lower BP. These two actions may occur in concert, and to varying degrees, each may be responsible for hypotension and the resulting transient LOC.^10^, ^11^ In cases in which cardioinhibition is the primary mechanism causing syncope in IA, and seizure freedom cannot be obtained by conventional epilepsy treatments, cardiac pacing may be beneficial.^7^, ^10^, ^11^ However, several reports suggest that syncope in IA may also be principally the result of vasodepression (i.e., vasodilatation); this may explain why pacing sometimes fails to prevent syncope recurrences.^8^, ^9^, ^12^

Disentangling the relative effects of cardioinhibition and vasodepression requires continuous BP measurement during the evolution of IA,^13^ a tool that is lacking with current routine video‐EEG recordings. However, we hypothesized that by analyzing the relative timing of the onset of syncope versus the beginning of asystole, we could provide insight into one aspect of the puzzle.^10^ Specifically, if asystole starts after onset of syncope or within about 3 s before syncope (a period in which it is generally accepted that the brain has sufficient metabolic reserve),^13^, ^14^ cardioinhibition is unlikely to be the primary cause.^10^ Consequently, the current study examined the temporal relationship between IA initiation and syncope onset with the objective, based on the 3 s threshold, of estimating how often cardioinhibition was unlikely the primary syncope mechanism in IA, and thereby how often pacemaker implantation may be beneficial in IA refractory to conventional antiseizure therapy.

## 2 METHODS

**Figure 1:**
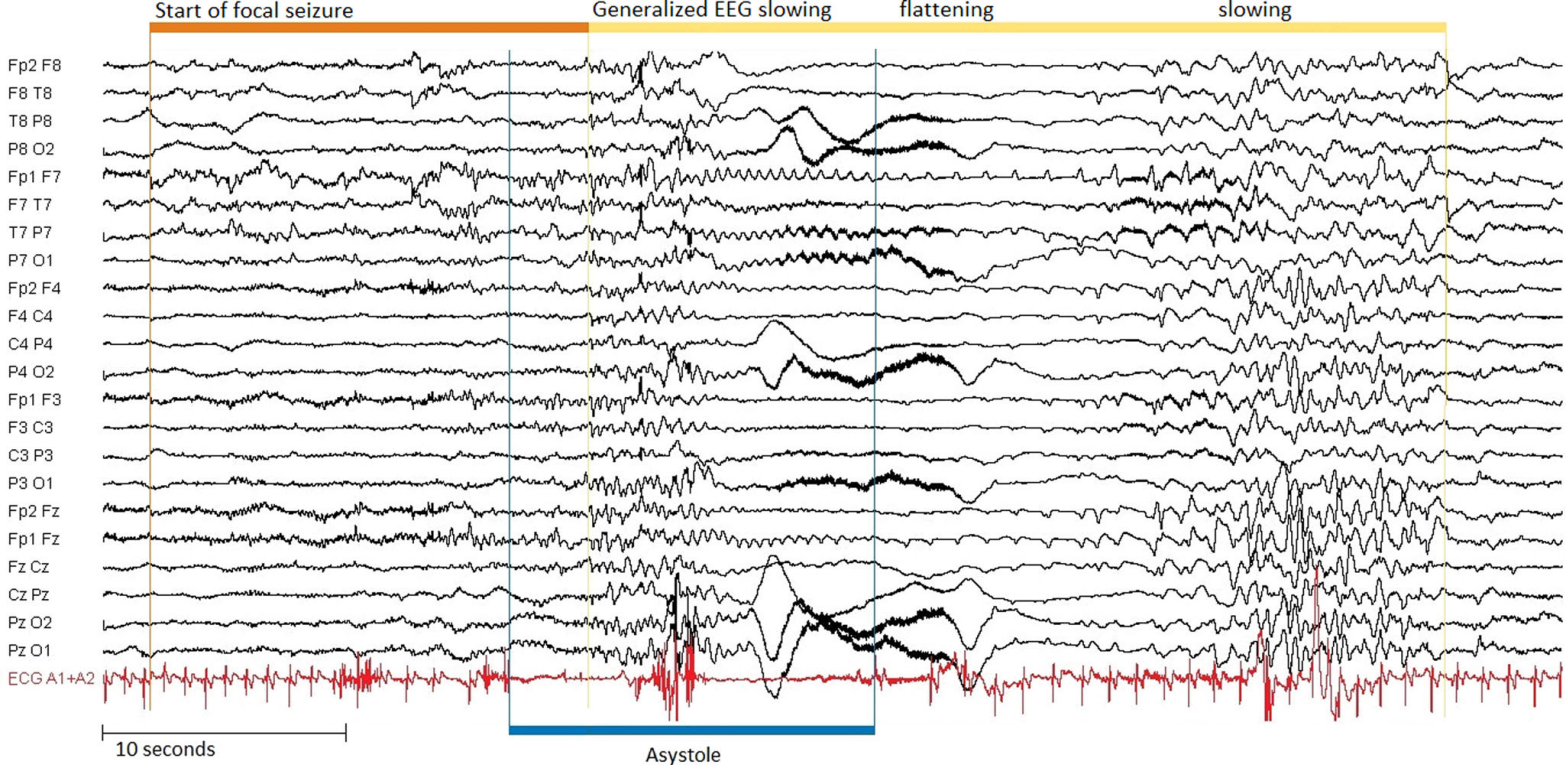
Typical EEG pattern during syncope in ictal asystole. Example of a 60 s EEG recording (filters 0.16–10 Hz, sensitivity 100 mV/cm) of a focal seizure originating in the left temporal lobe (orange bar) with ictal asystole (blue bar; duration 15 s) followed by syncope (yellow bar; duration 34 s). Syncope coincides with a slow‐flat‐slow pattern in the EEG (yellow bar; duration 34 s)^15,16^

We searched video‐EEG databases of five participating centers (Stichting Epilepsie Instellingen Nederland; Department of Epileptology Bonn; National Hospital for Neurology & Neurosurgery, London; New York University, Department of Neurology; University Medical Center Utrecht, Department of Neurophysiology) for focal seizures with IA, simultaneously recorded on video and EEG. IA was defined as any R‐R interval of ≥3 s preceded by heart rate slowing coinciding with ictal activity on EEG. Recordings with continuous video, EEG and one or two ECG leads were included. Multiple seizures with IA per person could be included. For every included subject, we listed all recorded seizures to derive an indication of the percentage of IA recurrence. Three authors in pairs of two (Roland D Thijs + Sharon Shmuely or Roland D Thijs + Anouk van Westrhenen) examined all IA recordings and checked whether the events met diagnostic criteria. IA timing and duration were derived from the ECG signal. Video recordings were reviewed for clinical expressions of loss of muscle tone (e.g., head dropping) to determine syncope onset time^14^ and duration, and body position (standing, seated or supine) during IA onset. Both researchers were blinded to the EEG and ECG signal during video evaluation. When the onset of unconsciousness could not be reliably determined from the video (e.g., if the individual was supine throughout), the classical EEG pattern during syncope, that is, generalized EEG slowing and/or flattening, was used to time syncope (Figure 1).^15^, ^16^

We applied previously defined criteria to classify the temporal relationship of IA to syncope onset,^9^ creating the following groups: (A) asystole starting after syncope; (B) asystole starting ≤3 s before syncope; (C) asystole starting >3 s before syncope, and (D) asystole without syncope. We assumed that cardiac bradycardia could not have been the dominant cause of syncope in Groups A and B.^10^

Data are presented as means ± standard deviation or median and range where appropriate. Differences between groups were analyzed using *χ* ^2^ statistics for categorical and the Mann–Whitney *U* test for unpaired continuous, not normally distributed data.

The medical ethics committee of the Leiden University Medical Center declared that the Medical Research Involving Human Subjects Act (in Dutch, the “WMO”) did not apply to this study as all data were acquired during routine clinical care. The data underlying this article cannot be shared publicly for the privacy of individual subjects. The data will be shared on reasonable request to the corresponding author.

## 3 RESULTS

We identified 38 focal seizures with IA in 29 individuals (17 male, median age: 41 years [range: 15–71 years]) who underwent evaluation from May 2001 to August 2018. Six had more than one seizure with IA (Table 1). As expected from a previous study,^17^ the risk for IA recurrence was relatively high and amounted to 27% in those who had had IA but who also had more than one recorded seizure. Syncope onset and end could not be determined using video in five seizures; in another seven seizures, only syncope end could not be determined. In these 12 cases, we used the EEG to determine syncope timing.

**Table 1:** Characteristics of included individuals

Group	Individual no.	Age/sex	Epilepsy etiology	Seizure type, onset zone	Total no. of recorded seizures	% IA recurrence^a^	IA duration (s)	Syncope duration (s)	Time between start IA and start syncope (s)	Body position	PM	FU duration	Syncope recurrence during FU
**B**	15	63/M	Structural	FIA, bitemporal	3	0	6	31^b^	2	Seated	No	5 years	No (seizure free after epilepsy surgery)
	22	58/M	Unknown	FIA, temporal L	1	_	15	31	3	Supine	No	5.5 years	No (seizure free with AED)
**C**	1	61/F	Infectious	FIA, temporal L	1	_	30	29	15	Supine	Yes	9 years	No
	2	50/F	Structural	FA, extratemporal R	15	13	16	Na^c^	6	Supine	Yes	14 years	No
	3	41/M	Unknown	FIA, temporal L	1	_	24	29	10	Supine	Yes	8.5 years	No
	5	41/F	Unknown	FIA, temporal R	1	_	14	12	10	Supine	Yes	3 years	No (seizure free)
	6	71/M	Unknown	FA, temporal L	1	_	20	11	15	Supine	Yes	2 months	No
	8	54/M	Infectious	FIA, temporal L	1	_	5; 3^d^	15	11	Seated	Yes	1 year	No (seizure free)
	9	15/F	Unknown	FIA, temporal L	1	_	27	33	10	Supine	Yes	3 years	No
	10	21/F	Unknown	FIA, temporal R	2	0	26	37	9	Seated	Yes	8 years	No
	11	23/M	Structural	FIA, temporal R	1	_	20	27	17	Supine	No	3 years	Seizure recurrence without syncope 6 months after epilepsy surgery
	12	36/F	Unknown	FIA, temporal L	1	_	29	25	10	Supine	Yes	10 years	No
	16	41/M	Unknown	FIA, temporal R	1	_	32	43	9	Supine	Yes	None	Na
	18	57/F	Immune	FIA, bitemporal	2	100	26	33	10	Seated	Yes	4 years	Yes, but fewer falls after PM implantation
				FIA, temporal L			19	15	10	Seated			
	19	33/M	Unknown	FIA, temporal L	1	_	13	14	10	Seated	No	None	Na
	23	27/M	Structural	FIA, temporal L	1	_	20	30	10	Seated	No		No (seizure free after epilepsy surgery)
	24	56/F	Unknown	FIA, temporal R	2	0	17	20	12	Seated	Yes	8 years	No
	25	28/M	Unknown	FIA, temporal R	2	0	12	11	10	Seated	Yes	11.5 years	Seizure recurrence without syncope after epilepsy surgery
	27	48/M	Structural	FIA, temporal L	2	0	11	23	4	Seated	Yes	8 years	No (seizure free after epilepsy surgery)
	28	27/M	Unknown	FIA, extratemporal R	2	0	23	26	9	Supine	Yes	2 years	No
	29	41/M	Structural	FIA, temporal L	2	0	24	37	9	Seated	Yes	4 years	No (seizure free after epilepsy surgery)
**D**	2	50/F	Structural	FA, extratemporal R	15	13	3; 3; 3; 3^d^	‐	‐	Supine			
				FA, extratemporal R			4; 3^d^	‐	‐	Supine			
	4	49/M	Structural	FBTC, temporal L	3	50	9	‐	‐	Seated			
				Focal onset tonic,^e^ temporal L			8; 4^d^	‐	‐	Supine			
	7	28/F	Unknown	FIA, extratemporal L	4	0	3	‐	‐	Supine			
	13	22/F	Unknown	FIA, temporal R	3	0	8	‐	‐	Supine			
	14	40/M	Unknown	FIA, temporal R	2	0	9	‐	‐	Supine			
	17	47/F	Unknown	FIA, temporal R	1	_	4; 4^d^	‐	‐	Supine^f^			
	20	16/M	Unknown	FIA, bitemporal	2	100	5	‐	‐	Supine			
				FIA, temporal R			5	‐	‐	Supine			
	21	16/M	Unknown	FIA, temporal R	4	100	8; 3^d^	‐	‐	Supine			
				FIA, bitemporal			9	‐	‐	Supine			
				FIA, bitemporal			8	‐	‐	Supine			
				FIA, temporal L			5; 8^d^	‐	‐	Supine			
	26	21/F	Structural	FIA, temporal L	2	100	5	‐	‐	Seated			
				FIA, temporal L			5	‐	‐	Seated			

*Note*: Characteristics of included individuals with IA, divided per group. Group (B) asystole starting ≤3 s before syncope; (C) asystole starting >3 s before syncope and (D) asystole without syncope.

Abbreviations: B, bilateral; F, female; FA, focal onset aware; FBTC, focal to bilateral tonic‐clonic; FIA, focal onset impaired awareness; IA, ictal asystole; L, left; M, male; Na, not available; No., number; PM, pacemaker; R, right; s, seconds.

^a^ IA recurrence during video‐EEG monitoring defined as a percentage of recurrent seizures with IA in those who had more than one recorded seizure.

^b^ Possibly facilitated by β‐blocker.

^c^ Syncope end could not be determined using video. The EEG recording was not available.

^d^ These numbers reflect multiple asystolic events within one seizure.

^e^ Awareness could not be assessed, because the individual was covered by a blanket.

^f^ Closed curtain blocked the view of the individual at the beginning of the seizure. When the curtain was moved aside, the individual was lying down.

**Figure 2:**
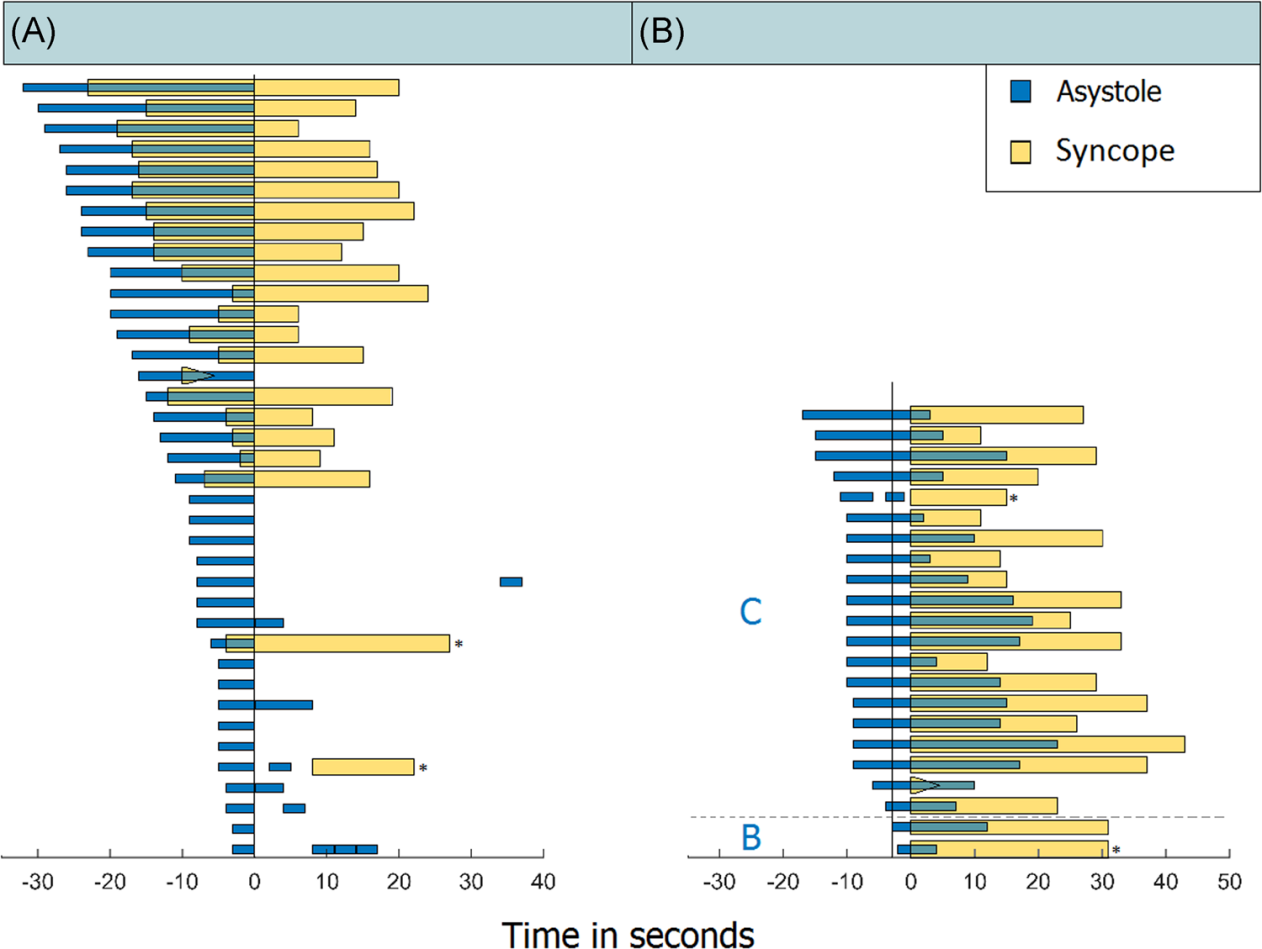
Relative timing of ictal asystole (IA) to onset of syncope. The horizontal bars represent one seizure each; blue bars indicate asystole and yellow bars the duration of loss of consciousness (LOC). In one case syncope end could not be determined using video and the EEG recording was not available (yellow triangle). (A) All 38 IA events sorted according to their duration in seconds and aligned to the end of asystole. Note that syncope was rare in seizures with short asystole (lower bars) but occurred in all those with an asystole duration ≥10 s. (B) All 22 syncopal events sorted by their time difference in onset of asystole and syncope, and aligned to the beginning of LOC. The vertical line identifies the threshold of 3 s before syncope. The horizontal dotted line separates seizures in which asystole started ≤3 s before syncope (Group B) and >3 s before syncope (Group C). *Two cases with an asystole <10 s and syncope in group A, one in group B and one in group C.

The median IA duration was 8 s (range: 3–32 s) and the mean syncope duration was 25 ± 9.4 s (Figure 2A). In seven seizures, there was more than one asystole period within one seizure. Two individuals experienced these sequential IAs in two different seizures, suggesting that some individuals might be more prone to this phenomenon (Table 1, nos. 2 and 21).

Syncope occurred in 22 out of 38 seizures with IA (58%). All IA events preceded syncope (Figure 2B); consequently, none was classified as belonging to Group A (0%). In two seizures, IA started ≤3 s before syncope (Group B, 5%) and in 20 seizures IA started >3 s before syncope (Group C, 53%). Sixteen seizures (42%) fell in Group D (asystole without syncope).

Seizures with syncope had a longer asystole than those without (median duration: 20 [range: 5–32] vs. 5 [range: 3–9] s; *p* < .001). Syncope occurred in all 20 IA events of ≥10 s and in only 2 of 18 IA events of <10 s. In only one of these events did the temporal sequence of IA and syncope meet the criteria of Group B (31 s of syncope, starting <3 s after onset of an IA lasting only 6 s), while another presented with two short sequential IA events (5 and 3 s) followed by 15 s of syncope >3 s after IA onset (Figure 2, marked by asterisk). The temporal sequence in both cases argues against a mainly cardioinhibitory mechanism as the dominant cause of syncope. One individual (Table 1, no. 2) had two seizures including multiple consecutive IAs of <10 s without syncope (Group B), as well as one seizure with asystole of 16 s followed by syncope, starting 6 s after IA onset (Group C).

Finally, syncope occurred more often in those patients who were seated compared to those who were lying down at the start of IA (11/14, 79% vs. 11/24, 46%; *p* = .049). The latter supports the view that in people with IA, the threshold for syncope is impacted by posture‐related effects on BP.

The 22 subjects experiencing IA with syncope had a median follow‐up period of 5.3 years [range: 2 months–11.5 years], with two lost to follow‐up. Sixteen out of nineteen subjects (84%) with asystole starting >3 s before syncope (Group C) received a pacemaker during follow‐up. Of the remaining three, one subject was seizure‐free after epilepsy surgery, another experienced only seizures without syncope after epilepsy surgery, and the last one was lost to follow‐up. Only one subject from Group C experienced syncope recurrence after pacemaker implantation (6%).

## 4 DISCUSSION

### 4.1 Main findings

This study provides three main findings. First, we found that in most IA cases the onset of asystole occurred early enough before syncope onset that cardioinhibition may have been the dominant syncope mechanism. Conversely, only in a minority of cases did IA start too close to the onset of syncope (≤3 s) to have been the primary cause. In this smaller group of individuals, pacemaker implantation may not prevent syncope as vasodepressor hypotension may have already progressed sufficiently to result in syncope. Second, syncope often lasted longer than did the asystole, suggesting that another factor may have become operational in sustaining LOC. The latter factor may have been later onset or slower evolution of a vasodepression component during the event. Although the numbers are small, within one person multiple IA events exhibited the same presumptive dominant syncope triggering mechanism (i.e., cardioinhibition or vasodepression). This observation tends to lend support to the expected pacemaker utility in patients with cardioinhibition detected.

Finally, our long‐term follow‐up results show that pacemaker treatment was effective to prevent or reduce syncope recurrence in all cases in which syncope started >3 s after IA onset (Group C).

### 4.2 Pacemaker therapy in IA

IA is most commonly associated with seizures arising in the temporal lobe or nearby insula region. Stimulation of the latter has, in particular, been associated with triggering spells similar to vasovagal syncope.^6^ In any case, the primary treatment of IA is optimizing seizure control by antiseizure medication or epilepsy surgery.^5^, ^7^, ^8^, ^9^ In terms of drugs, a number of agents are readily available and are generally well tolerated.^18^ Additionally temporal lobe resection surgery has proved generally effective. However, if seizure freedom cannot be obtained, pacemaker implantation may be considered, but guidelines are lacking.^8^, ^9^ Case series suggest that pacemakers may reduce falls and injuries, but these observations are based on potentially unreliable diary data; large follow‐up studies are lacking.^12^, ^19^, ^20^ Furthermore, if pacemaker treatment is considered, careful pacemaker programming is important as one recent case report has highlighted the possibility that excessive pacing may unintentionally delay seizure termination, by maintaining cerebral perfusion and prolonging IA.^21^

### 4.3 Syncopal LOC mechanism

Cardiac standstill causes syncope when the duration of circulatory arrest exceeds the cerebral ischemic anoxia reserve time.^14^ The anoxia reserve time may vary among individuals from 4 to 15 s with an average duration of 5–6 s.^14^ Consequently, it is reasonable to expect that an isolated cardiac standstill of less than 3 s cannot lead to syncope.^10^, ^16^, ^19^ Using the 3 s threshold, we concluded that cardioinhibition was the dominant pathomechanism for syncope in the majority of our cohort. However, whether vasodepression ensued later or more slowly during the episode in some patients, thereby representing a differential effect of asystole on syncope onset and end, or an additional process, remains an unknown in need of future study.

### 4.4 Impact of posture on syncope

Upright body position appeared to contribute to syncope susceptibility in our IA patients. This finding suggests a role played by gravity; presumably, upright position accelerated cerebral hypoperfusion whether due to cardioinhibition or vasodepression. Unfortunately, we did not have access to BP data in our cases, but other reports tend to support this contention.^10^, ^16^ Continuous BP recordings in two people with temporal lobe epilepsy and ictal bradycardia in the supine position illustrated a progressive BP decrease before bradycardia in one and a BP decrease with concomitant bradycardia in the other.^20^ Another case report on temporal lobe epilepsy and recurrent ictal syncope after pacemaker implantation for IA, demonstrated symptomatic hypotension during a focal seizure in the supine position, despite pacemaker activation.^12^ The latter finding suggests that seizure‐induced vasodepression can cause syncope on its own.

A study on asystole and LOC timing in tilt‐induced reflex syncope revealed a lower mean arterial pressure (MAP) in syncope occurring ≤3 s after asystole than in later onset syncope^10^; this suggested a major role of vasodepression causing syncope in these cases. Low MAP, however, was also observed in some asystole events occurring >3 s before syncope,^9^ raising the possibility that the contribution of vasodepression to the occurrence of syncope may be underestimated using this approach. Perhaps vasodepression takes longer to evolve and acts less to start the event than to prolong it as suggested earlier.

### 4.5 Limitations

Interpretation of our findings is limited by a number of factors. First, the ability to detect syncope within 3 s of onset of asystole may be questioned. In this regard, we set up a method in which groups of experienced yet independent observers determined the timings and differences were adjudicated. Second, inferences regarding the possibility that vasodepression may extend the syncope period beyond the duration of asystole cannot be substantiated by direct BP measures, and remains to be reassessed in future studies. Finally, while the overall number of patients was relatively large in terms of published IA studies, the number of cases with multiple episodes was small. These numbers only include those seizures that are recorded on video‐EEG during a short clinical stay, thus only reflecting a snapshot. Therefore, conclusions regarding the consistency of pathophysiology within an individual warrant further study.

## 5 CONCLUSION

Cardionihibition appears to play an important role in syncope associated with seizure‐induced IA; in only a few cases is vasodepression the dominant triggering mechanism. Consequently, in most IA cases, when conventional therapy has not adequately prevented syncope due to seizure recurrences, cardiac pacemaker therapy is likely to prove helpful.

## Data Availability

The data that support the findings of this study are available on request from the corresponding author. The data are not publicly available due to privacy or ethical restrictions.
